# Temporal sampling in vision and the implications for dyslexia

**DOI:** 10.3389/fnhum.2013.00933

**Published:** 2014-02-17

**Authors:** Kristen Pammer

**Affiliations:** The Research School of Psychology, The Australian National UniversityCanberra, ACT, Australia

**Keywords:** reading, dyslexia, vision, temporal coding, oscillation, synchronisation, review

## Abstract

It has recently been suggested that dyslexia may manifest as a deficit in the neural synchrony underlying language-based codes (Goswami, 2011), such that the phonological deficits apparent in dyslexia occur as a consequence of poor synchronisation of oscillatory brain signals to the sounds of language. There is compelling evidence to support this suggestion, and it provides an intriguing new development in understanding the aetiology of dyslexia. It is undeniable that dyslexia is associated with poor phonological coding, however, reading is also a visual task, and dyslexia has also been associated with poor visual coding, particularly visuo-spatial sensitivity. It has been hypothesized for some time that specific frequency oscillations underlie visual perception. Although little research has been done looking specifically at dyslexia and cortical frequency oscillations, it is possible to draw on converging evidence from visual tasks to speculate that similar deficits could occur in temporal frequency oscillations in the visual domain in dyslexia. Thus, here the plausibility of a visual correlate of the Temporal Sampling Framework is considered, leading to specific hypotheses and predictions for future research. A common underlying neural mechanism in dyslexia, may subsume qualitatively different manifestations of reading difficulty, which is consistent with the heterogeneity of the disorder, and may open the door for a new generation of exciting research.

Developmental Dyslexia is a cognitive learning difficulty where a child demonstrates a specific problem in reading, with no obvious cause. A “definition by exclusion,” it refers to a child that has experienced normal teaching and learning environments, has had normal social experiences, has no other comorbidities, and has a normal IQ, yet still demonstrates a specific reading difficulty (Scanlon, 2012). In reality, a dyslexic child quite often demonstrates comorbidities, or experiences impoverished social environments for example, and a child with a low (or high) IQ can still present as dyslexic. However, the definition by exclusion is important for researchers when trying to isolate biological bases for dyslexia, as it allows them to be more confident that the cognitive disorder the child is presenting with, is in fact likely to be dyslexia. Moreover, dyslexia is not just a childhood problem. Many adults who have suffered from developmental dyslexia as children never develop good reading skills (Hatcher et al., 2002). Those who do compensate for their reading difficulty and become good readers, invariably suffer from residual difficulties such as poor spelling and poor phonological coding (Lindgren and Laine, 2011).

The majority of children presenting with dyslexia demonstrate problems with phonics, where “phonics” is generally characterized as sensitivity to the subtle sounds of language. Tests of phonological awareness tend to be tests of how well an individual can understand, segment and manipulate speech and language sounds. Poor phonological coding precedes subsequent poor reading: most dyslexic children demonstrate some kind of difficulty in phonological coding, poor phonological coding remains when children grow into adulthood and develop compensated reading skills, and explicit training in phonics is the best strategy available with regard to a treatment for dyslexia (refer to Snowling, 2000 for a review). However, in regards to the latter point, phonics training is the best form of remediation as indicated by current evidence, but this could also be because research looking at other types of training such as visual training (e.g., Franceschini et al., 2013) or training grapheme-phoneme correspondences based on grain size (e.g., Kyle et al., 2013) is in its infancy. Thus dyslexia is often considered a form of language disorder as its basic aetiology may be in the form of deficits in auditory coding that make it difficult to develop stable phonological-graphemic relationships.

In addition to phonological coding, a large amount of research over the last 40 years has also demonstrated that many dyslexic readers have subtle visual deficits. Historically, dyslexia was considered a visual deficit, in the form of congenital word blindness; however, that dyslexic readers consistently demonstrated normal visual acuity, challenged the old “visual deficit” hypothesis in favor of deficits in phonological coding. Nevertheless, subsequent evidence suggests that many dyslexic readers appear to suffer from a deficit in coding visual information that is specific to the dorsal (or magnocellular) visual pathway (e.g., refer Stein, 2001; Pammer and Vidyasagar, 2005 for reviews).

## The magnocellular deficit theory of dyslexia

At the subcortical level, the visual system consists of at least two pathways, magnocellular and parvocellular pathways, which carry visual information from the retina, through separate layers of the lateral geniculate nucleus (LGN) and project to distinct layers of primary visual cortex (V1). The two pathways run parallel to each other and consist of neurons which differ not only anatomically but also physiologically (Galaburda and Livingstone, 1993; Merigan and Maunsell, 1993; Zeki, 1993), suggesting that the they are specialized for processing different kinds of visual information. The magnocellular pathway demonstrates a high degree of sensitivity to low contrast, low spatial frequency, high temporal frequency, and achromatic visual information (Merigan and Maunsell, 1993). Consisting of large heavily myelinated neurons with fast conduction velocity, the magnocellular pathway responds maximally to rapid temporal changes, with magnocells responding at stimulus onset/offset rather than throughout the entire stimulus presentation. Conversely, the parvocellular pathway consists of small neurons that are sensitive to high spatial frequency, low temporal frequency, and color information, demonstrating sustained response activation throughout the entire duration of the stimulus (Goodale and Milner, 1992; Merigan and Maunsell, 1993).

From V1 the anatomical and functional dissociation of the magnocellular and parvocellular pathways becomes less clear, with visual information from the two streams interacting considerably as they project to extrastriate visual areas (Ferrera et al., 1992; Merigan and Maunsell, 1993; Nealey and Maunsell, 1994; Vidyasagar et al., 2002). Anatomical data indicates that the magnocellular and parvocellular systems may converge as early as layer 4 of V1 (Yabuta and Callaway, 1998), suggesting that higher order dorsal processing may not be entirely indicative of lower level magno functioning. Nevertheless two distinct cortical streams, the dorsal and ventral streams respectively, exist (Benardete et al., 1992; Zeki, 1993). The ventral (or “what”) stream receives both magnocellular and parvocellular inputs as it projects to the inferotemporal cortex, an area specialized in extracting details relating to an object's shape and color (Zeki, 1993). This is in contrast to the magnocellular dominated dorsal (or “where”) stream, which passes through V5 before projecting to the posterior parietal cortex, a selective spatial attention area specialized for processing the location of objects in space. Thus the dorsal pathway is considered to be responsible for visual qualities such as spatial awareness and movement, while the ventral pathway is considered to be responsible for qualities such as color processing and visual detail.

It is thought that dyslexic readers demonstrate difficulties transmitting visual information that is carried by the dorsal/magnocellular pathway. Here “dorsal” and “magnocellular” are somewhat different in that “magnocellular” refers to the cells that make up the dorsal visual pathway, and the dorsal pathway actually contains a small proportion of both parvocells and koniocells. However in the literature this distinction has frequently been blurred, and for the sake of brevity I will use “dorsal/magnocellular” to refer to theories and research that refer to both or either terminology. This Theory has become known as the “magnocellular deficit” hypothesis (Stein, 2001). Why the dorsal pathway is impaired in dyslexia, remains open to speculation, but may lie in the possibility that developmentally, the dorsal pathway is more vulnerable than the ventral pathway (Braddick et al., 2003). Numerous behavioral studies have demonstrated that; dyslexic readers are less sensitive to visual information that is carried by the dorsal/magnocellular pathway (e.g., Martin and Lovegrove, 1987; Pammer and Wheatley, 2001; Wright and Conlon, 2009), that sensitivity to dorsal/magnocellular tasks exist in children at risk for reading difficulties before they learn to read (Kevan and Pammer, 2008), that dorsal /magnocellular deficits predict subsequent reading ability in pre readers at risk for reading impairment (Boets et al., 2008; Kevan and Pammer, 2009), and that dorsal/magocellular sensitivity is correlated with reading ability (Witton et al., 1998; Pammer and Kevan, 2007). There is also good neurophysiological evidence to support a deficit in the dorsal/magnocellular pathways in dyslexic readers (Eden et al., 1996; Demb et al., 1998; Jednorog et al., 2011). Yet despite deficits in the dorsal pathway, visual coding in the ventral/parvocellular pathway in dyslexic readers remains normal. There is also substantial evidence to suggest that dyslexic readers have deficits in other sensory domains, such as auditory (refer to Hämäläinen et al., 2013 for a recent review) and motor processes (e.g., Thomson et al., 2006; Thomson and Goswami, 2008), prompting the suggesting that dyslexia might be a disorder that encompasses sensory systems more globally (Stein and Walsh, 1997).

However the view that dyslexic children demonstrate sensory coding deficits is not universal. For example, auditory processing deficits have not been found in all dyslexic children (Hill et al., 1999; Edwards et al., 2004; White et al., 2006). Halliday and Bishop (2006) compared dyslexic, normal readers and children with sensory hearing loss on auditory frequency modulation thresholds, they demonstrated that an amplitude modulated signal disrupted both low and high frequency coding, but only the children with sensorineural hearing loss differed from the normal readers, with no difference found between normal and dyslexic readers. Similarly, in some cases no differences have been found between dyslexic and non-dyslexic readers in the perception of speech signals (Messaoud-Galusi et al., 2011). Similar discrepant results have been found for visual processing (Ben-Yehudah et al., 2001; Stuart et al., 2001; Amitay et al., 2002; Olson and Datta, 2002; Ramus et al., 2003; White et al., 2006), where dyslexic readers have not been found to be different from normal readers. Refer to Skottun for comprehensive, alternative reviews of the magnocellular literature (e.g., Skottun, 2005; Skottun and Skoyles, 2007, 2008). Ramus et al. (2003) has suggested that sensory deficits may be characteristic of specific groups of dyslexic readers, such that deficits in different domains may be characteristic of different behavioral manifestations. This notion will be revisited in the “questions and hypotheses” section of this paper.

One important question regarding visual coding and dyslexia is to understand the link between dorsal/magnocellular processing, reading, and dyslexia. Because dorsal/magnocellular processing is not intuitively a natural candidate to support reading skill (its area of expertise is in coding spatial information, movement, and contrast), this link is not obvious. Some of the specific visual tasks that dyslexic readers have difficulties with, include: orienting attention (Facoetti et al., 2001, 2006), focusing attention (Facoetti et al., 2000, 2003), scanning cluttered environments (Williams et al., 1987; Vidyasagar and Pammer, 1999; Sireteanu et al., 2008), and coding the locations of letters within words (Cornelissen et al., 1998). One interpretation of this evidence is that the dorsal/magnocellular pathway is important in the reading process by virtue of its role in attention (Hari et al., 1999; Iles et al., 2000; Vidyasagar and Pammer, 2010; Moores et al., 2011), specifically in the spatial coding and binding of letter and word features, letters within words, and directing saccadic movements across the page (Vidyasagar, 1999, 2004; Vidyasagar and Pammer, 1999). Problems with these processes would make it difficult for a young reader to generate stable visual representations of words, and move the eyes in a way to access the important visual qualities of text.

The notion that visual deficits in dyslexia may occur as a consequence of difficulties in binding the visual components of letters and words is consistent with what is known about the cortical processing of other visual objects. Object recognition is dependent on different parts of the cortex “binding” or synchronizing information to provide a coherent whole. For example, identifying a red bird flying from a tree requires at its very basic level, identifying the color of the bird, and the color of the leaves, identifying the bird-features and the tree-features, extracting the bird-object from the tree-object, and the movement of the bird, as distinct from the movement of the tree. This simple percept requires highly sophisticated cortical binding. For example, color needs to be bound to the locations of the various objects in space, which needs to be bound with the movement information. Moreover, all of this occurs within tens of milliseconds, and the visual system is extremely good at it; we would rarely—in the above example—perceive a green bird and red tree, suggesting that there are highly accurate and robust neural networks that communicate their information across time and space. Reading may be seen as an extension of this sophisticated cortical binding. Natural reading requires binding similar temporal and spatial information; letter features are to be bound into coherent letters, which are then placed within the correct locations within words, such that they can be identified, and the eye guided to the next location. Because the eye is moving very quickly across a page—fixations and saccades are in the order of 20 and 200 ms respectively—the spatio-temporal synchrony necessary to extract the letter, word, and sentence objects is really astounding.

However, like natural object recognition, these bottom-up processes do not occur in a linear, isolated fashion, but rather, are supported and sustained by top-down influences that facilitate recognition. For example, we demonstrated that “higher-level” cognitive functions in the reading network, such as language processing, are active within a few hundred milliseconds of the start of visual coding in the visual cortex. This was followed by both visual and language processing occurring in a dynamic, cascaded way, featuring feed-forward and feedback information flow (Pammer et al., 2004). This supports the notion that reading skills are dependent on a dynamic synthesis of both bottom-up and top-down information flow.

Thus, like other forms of object recognition, visual coding of text requires large populations of neurons to be synchronizing and synthesizing information extremely quickly, over disparate cortical areas to form coherent percepts.

## Temporal sampling

Neuronal firing has a stochastic element, showing in their discharge, a large amount of variability and seemingly random firing patterns (e.g., Wang, 2010). However, behavior is not dependent on single cells firing at random, but rather on the coordinated, synchronous firing of thousands of cells in a neuronal population. That the brain demonstrates rhythmic discharge variations within neuronal populations has been known since the 20's with the first recordings of the alpha rhythm (Berger, 1929) and has resulted in hundreds of papers dedicated to understanding how and why such cortical rhythms occur. Indeed it is likely that unlocking the secrets of cortical rhythms will unlock many of the secrets of the brain.

In the current context, I will consider only rhythmic neural oscillations at the macroscopic level. Although oscillations have been observed for many years at the single-cell level (e.g., Hodgkin and Huxley, 1952; Llinas et al., 1991; Llinas and Steriade, 2006), the rhythmic cortical activity observable using EEG or MEG occurs when large populations of neurons synchronize to produce oscillations with a common frequency, amplitude and phase (Hämäläinen et al., 1993). In some cases, populations of neurons decouple from a common oscillation to synchronize at a different frequency or amplitude. This is referred to as Event Related Desynchronisation (ERD), and Event Related Synchronisation (ERS) occurs when local cell populations synchronize to form a coherently oscillating population (Pfurtscheller and Lopes da Silva, 1999). Moreover, populations of neurons in different areas of the brain can synchronize their oscillatory activity, which has been hypothesized to reflect cortical communication (Singer, 1999, 2009; Wang, 2010; Thut et al., 2012). It is this synchronisation of oscillatory behavior that is believed to underlie cortical binding, perception, cognition and behavior (Singer, 1999; Engel and Singer, 2001; Wang, 2010; refer to Siegel et al., 2012; Thut et al., 2012 for recent reviews).

Although it is assumed that cortical rhythms are causally related to behavior, it is possible that such cortical rhythms are simply epiphenomenal to information processing and behavior. However, there are a number of brain rhythms that have been associated with different cognitive or behavioral states, although there is a great deal of fluidity around the notion that particular cortical rhythms = cognitive function, and all the oscillatory rhythms that are generated by the brain have been demonstrated in one way or another with most cognitive functioning. Nevertheless, there is some broad consistency in the literature regarding some frequency ranges and cognitive/perceptual functioning. For example, theta rhythms (4–8 Hz) have been associated with maze navigation (Caplan et al., 2003; Kahana et al., 1999), episodic memory (Lega et al., 2012; Burke et al., 2013) and working memory (Sauseng et al., 2009; Moran et al., 2010). The alpha rhythm is one of the primary brain rhythms and frequently associated with various aspects of perception and cognition (e.g., Pfurtscheller and Klimesch, 1991). Beta rhythms (15–30 Hz) have been associated with motor preparation (Alegre et al., 2006; Cheyne et al., 2012) and control (e.g., Salmelin et al., 1995; Stancák and Pfurtscheller, 1996), and gamma rhythms (30–80 Hz) appear to be an index of attention (Jensen et al., 2007), feature binding (Tallon-Baudry et al., 1997) and object recognition (Tallon-Baudry and Bertrand, 1999; Martinovic et al., 2008; Friese et al., 2012). Some frequencies have been directly associated with changes in perception, for example, Fries et al. (2001) demonstrated that a gamma response in the visual cortex increases in response to attending to a behaviorally relevant stimulus. Connectivity at specific frequencies has also been demonstrated to reflect behavior. We demonstrated (Kujala et al., 2007) that unique areas of the brain synchronized at 8–12 Hz (alpha range) in response to different reading requirements. In this study, participants were presented with continuous text presented at rates that made comprehension easy, effortful, very difficult (only the general gist of the story was apparent), or impossible (random text). Left hemisphere cortical activations consistent with a reading network were activated at 8–12 Hz in a dynamic way that reflected the cognitive requirements of the reading task. Similarly, Hummel and Gerloff (2005) required participants to perform a visuo-tactile integration task, where a braille-like pattern was to be matched to a visual pattern on a computer screen. Long-range coherence between visual and motor areas, at the alpha frequency increased with better performance on the visuo-spatial integration task.

There are many examples of changes in oscillatory power or frequency in response to changes in information processing (refer Siegel et al., 2012 for a recent review), supporting the proposal that cortical oscillations, and the synchronisation of oscillations may represent a biological mechanisms for perception and cognition (e.g., Neuper and Pfurtscheller, 2001).

## Auditory temporal sampling in dyslexia

It has recently been suggested that poor phase-locking at low frequency theta and delta bands, (approximately 2–10 Hz), could be the proximal cause of dyslexia (Goswami, 2011). Phase-locking, also referred to as phase-synchrony (Lachaux et al., 1999), is the synchronisation of a brain signal at a specific frequency, reflecting the fact that the phase of the neural oscillation synchronizes with other neural oscillations, typically as fast evoked response to an external signal. Unstable auditory phase-locking at 2–10 Hz is believed to underlie the specific phonological impairments characteristic of dyslexia, and it has been suggested that such impairments may reflect a broader multi-sensory deficit, explaining some of the visual deficits also apparent in dyslexia (Goswami, 2011).

However, abnormal cortical oscillations in the auditory domain in dyslexia are not unique to theta or delta oscillations. For example, Lehongre et al. (2011) demonstrated abnormal auditory frequency dynamics in dyslexic readers in the gamma (25–35 Hz) frequency range. They presented dyslexic and normal readers with an auditory stimulus that was modulated linearly from 10 to 80 Hz. The associated auditory entrainment in the gamma range in normal readers was lateralized to the left hemisphere, specifically the superior temporal sulcus and planum temporale. They suggested that this neural signal is necessary for the fast formant transitions that occur in phonemic segregation. In dyslexic readers, however this gamma signal to the same stimulus was significantly reduced—providing a potential neurophysiological correlate of the common observation that dyslexic readers are impaired in phonological processing (Snowling, 2000). Moreover, dyslexic readers showed increased entrainment at 30 Hz in the right hemisphere—both compared to their left hemisphere, and compared to normal readers. This is also consistent with the evidence that dyslexic readers may develop compensatory right hemisphere reading networks, compared to normal readers (Leonard and Eckert, 2008), particularly for phonological output measures such as rapid automatized naming which requires left and right hemisphere integration (Eden et al., 2004). In addition, dyslexic readers show abnormally strong entrainment in the high gamma range (50–70 Hz) in the planum temporale of both hemispheres compared to controls.

Using sentences presented auditorily, Han et al. (2012) demonstrated that normal readers synchronize (normalized phase locking) high frequency gamma band (30–45 Hz) information bilaterally in the left and right auditory cortex, such that phonologically similar target words resulted in increased phase-locking, while phonologically dissimilar words resulted in decreased phase locking. Dyslexic readers however showed the opposite pattern of synchronisation. Since phonologically similar words require a more sophisticated analysis of phonemic information in order to distinguish between a “sensible” word, and a phonemically similar foil, the findings suggest better phonemic segmentation for heard words in normal readers compared to the dyslexic readers.

Clearly then cortical oscillatory activity in the auditory domain differs in dyslexia compared to normal reading. According to the temporal sampling hypothesis proposed by Goswami (2011), abnormal neural oscillatory activity may be responsible for dyslexia as a consequence of poor theta synchronisation to the sounds of language. There is also evidence for abnormal oscillatory activity in higher frequencies for dyslexic readers in response to auditory processing, which may also contribute to poor auditory temporal coding of language signals, making it difficult to develop good reading skills. However, reading is foremost a visual process, and dyslexia has also been associated behaviorally and physiologically with deficits in the dorsal visual pathway. Therefore, is there evidence for a visual correlate of the auditory temporal sampling hypothesis?

## Phase locking of speech and reading signals

**Figure 1:**
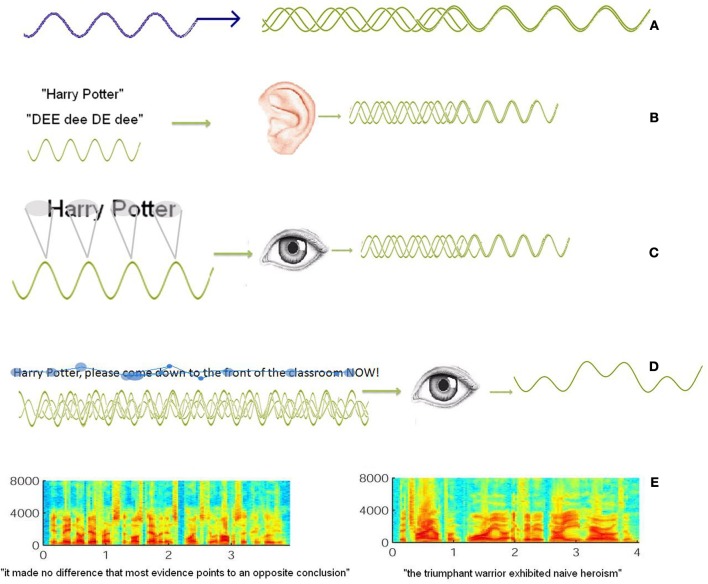
**(A)** Is an illustration of how oscillatory signals—either external, or internal, can entrain a cortical oscillation, such that networks of neurons synchronize to oscillate at a critical frequency. It has also been suggested that the rhythmic nature of spoken language, such as “Harry Potter” in “dee's” (Goswami, 2011) can entrain an oscillatory cortical signal **(B)**. Moreover, the sequential spatial coding of words when reading lends itself to temporal sampling at much the same frequencies **(C)**. However, **(D)** is an example of how when reading, the unpredictable nature of fixations, saccades and regression might make it difficult to generate a single stable oscillatory signal, which in turn might make it difficult to entrain an associated oscillatory signal in the visual cortex. Here, the blue dots represent fixations of varying length and regressions. Similarly, when listening to speech, sentences have different spectral energy, **(E)** the sentence on the left has a spectral signature that is much more regular than that on the right. Nevertheless, Luo and Poeppel (2007) demonstrated entraining in the auditory cortex to both sentences (reproduced with permission from Luo and Poeppel (2007), supplementary material. Elsevier).

Entrainment refers to oscillatory activity of neurons becoming synchronized with a repeated signal or perturbation (Pikovsky et al., 2003). External entrainment can occur by providing constant rhythmic stimuli such that neural assemblies phase-lock to the stimuli, synchronize their phase, and thus increase the neural signal (Thut et al., 2011). In this case, synchronizing and entrainment refer more-or-less to the same result—neural populations that have phase-locked to a signal and now oscillate at the same (or its harmonic Price and Ibbotson, 2001) frequency (refer to Figure 1A). One of the proposals of the temporal sampling hypothesis, is that auditory coding entrains to the temporal sampling of the speech signal—specifically its syllabic structure, and that this entrainment occurs at approximately the theta rhythm reflecting the temporal rhythm of syllabic structure (refer to Figure 1B). Dyslexia occurs when this phase-locking, entrainment and synchronisation are impaired, resulting in poor coding of speech, and thus a difficulty in generating stable language-graphemic representations. Using the same arguments, it should also be possible for the visual system to entrain a visual signal that is phase-locked into the visual sampling of text.

Like the auditory coding of the speech stream, visual coding of text requires sequential sampling of words and text (refer to Figure 1C), and this sampling rate is consistent with the sampling rate of speech, i.e., approximately 2–10 Hz. In reading, fixations occur approximately every 200 ms (Rayner, 1998), and understanding the text requires the very fast sampling and concatenation of information from fixations to form a continuous, understandable percept—much like understanding speech. Moreover, it has been suggested that the temporal-spatial sampling of text is controlled by the magnocellular/dorsal stream, which then gates sampled information to the ventral pathways and other higher order cognitive mechanisms (Vidyasagar, 1998, 1999). If then, similar temporal mechanisms exist in both speech perception, and reading, it is possible that a common mechanism underlies both, and this would predict that dyslexic readers would be less sensitive to some of the visual equivalents of auditory signal processing.

It has been demonstrated that dyslexic readers are less sensitive to visual stimuli of low *spatial* frequency (Lovegrove et al., 1980; Badcock and Lovegrove, 1981; Slaghuis and Ryan, 1999), but the above hypothesis would predict that dyslexic readers would be less sensitive to visual signals that are presented at a regular temporal frequency, in much the same way they are impaired at processing auditory signals presented at a regular temporal frequency (refer to Goswami, 2011 for a review). However, “temporal coding” in vision in dyslexia can mean quite different things, and the research findings are highly variable. One interpretation of temporal coding is the speed at which the visual system can code visual information, another is the ability for the visual system to detect rapidly changing temporal events (e.g., refer to Farmer and Klein, 1995; McLean et al., 2011), and the research literature rarely distinguishes between the two.

There is evidence to suggest that dyslexic children have difficulties in sequencing the temporal order of quickly presented stimuli (e.g., di Lollo et al., 1983; Brannan and Williams, 1988; Hari et al., 2001; Jaskowski and Rusiak, 2008; Liddle et al., 2009). These are typical Temporal Order Judgment (TOJ) tasks in which the participant is presented with a number of items (usually 2 or 3) in fast succession, and required to indicate their temporal order. The problem with TOJ tasks is that they can be quite difficult, and have quite a high memory load, so any differences between dyslexic and non-dyslexic readers could manifest from a number of different perceptual or cognitive causes. Similarly, the length of time it takes for a neural signal to decay in order to identify two discrete events, is Visible Persistence (VP). Dyslexic readers have been shown to require longer delays between signals (Badcock and Lovegrove, 1981; Slaghuis and Lovegrove, 1985; Lovegrove et al., 1986; Slaghuis and Ryan, 1999; Conlon et al., 2004), suggesting a longer period of neural persistence, although other studies have failed to find such a difference (e.g., Schulte-Körne et al., 2004). Temporal coding can also mean how quickly the visual system can deal with visual information i.e., processing speed. McLean et al. (2011) measured the temporal integration thresholds of the magnocellular and parvocellular pathways respectively. Here two isoluminant colored patches are alternated to “flicker.” The temporal resolution of the flicker increases until the color merges, but the flicker is still noticeable—this is the parvocellular resolution limit. If the temporal frequency is increased further, then the colors not only merge, but the perceptual flicker goes away—this is the magnocellular resolution frequency. In this study, dyslexic children showed lower resolution thresholds compared to normal readers in their magnocellular, but not parvocellular thresholds.

If temporal coding in a language framework is predicated on the rhythmic frequency modulation of speech, and if temporal coding in reading is the rhythmic saccadic sequencing of visual information, then Attentional Blink (AB) may be a useful candidate to conceptualize visual temporal coding, and has the added bonus of at least one theoretical framework that is based on rhythmic pulses of neural signals. AB is a task that involves an RSVP stream of distractor stimuli (such as letters) in which are imbedded two targets (such as numbers); T1 and T2. Perception of T2 decreases when the delay between T1 and T2 is between 200–400 ms. The Boost and Bounce theory of AB (refer to Olivers and Meeter, 2008 for a comprehensive discussion of this theory) suggests that AB reflects a continuous, rhythmic sequence of visual signals. In dyslexia then if this fast temporal signaling is impaired (slower, or longer, less defined for example), then it might predict a number of outcomes: dyslexic readers should show less T1-sparing and lower detection of T1 because of a more prolonged signal from the distractor the precedes it. Although the research findings are mixed in AB and dyslexia, this is in fact one common finding (e.g., Hari et al., 1999; Visser et al., 2004; Facoetti et al., 2008). The dip in the “blink” might also be prolonged, and/or shifted to the right in dyslexic readers because the signal from T1 also lasts longer, which is consistent with some findings (Hari et al., 1999; Visser et al., 2004; Facoetti et al., 2008), although others have failed to find this effect (e.g., Lallier et al., 2010). Others have found no differences between dyslexic and normal readers (Badcock et al., 2008; McLean et al., 2010). One of the problems about AB is that there are multiple ways in which the dip can be analyzed and reported, which makes it difficult to compare results.

For the purposes of this paper, “temporal coding” is probably best conceptualized as both processing speed and the ability of the visual system to deal with information presented quickly, such as at the rate of saccadic and fixation sequences, or even faster. As in the auditory domain, temporal coding here really refers to the ability of the visual system to effectively process quickly presented information. Here “quickly presented information” comes about either because stimuli is presented quickly in sequence, such as in an RSVP (IB) task, or the visual system engages in a sequential temporal sampling process such as when reading. Thus, as an RSVP task presents the visual system with fast discrete visual percepts, so does reading when the bottom-up data acquisition during a fixation is separated by a “dwell time” caused by the saccade. The differences between the temporal processing tasks above and reading, is that the temporal component in the above tasks is procedural, caused by the stimuli itself and the eyes are static, compared to reading, where the temporal component is mechanical, caused by the fixations, saccades and eye movements, while the stimuli is static. A summary of some of the research regarding temporal coding in dyslexia is in Table 1. However, an important distinction here is that this is behavioral research, where “temporal coding” refers to the ability to process quickly presented information. It is still unclear the degree to which these studies inform the neural process of temporal coding which refers to the synchronisation of neural signals.

**Table 1:** **A summary of some of the behavioral evidence regarding visual temporal coding in dyslexia**.

**Study**		**Task details**			**Outcome**	
**Attentional blink**		**Distracters**	**Targets**	**Timings**	**Magnitude of dip**	**Location of dip**
	Hari et al., 1999	Black letters	White letter	106 ms, no ISI	Dyslexics generally poorer at detecting the target	Shifted to longer durations for dyslexics
	Visser et al., 2004	Random dots	A shape (square, cross etc.)	40 + 60 ms ISI	Dyslexic generally poorer than controls, similar pattern as for reading matched controls	Shifted to longer durations for dyslexics compared to controls, same patters as for reading matched controls
	Facoetti et al., 2008	None. Only T1 and T2 were presented at varying intervals and each were masked	Letters, each had a pre and post-mask	T1 and T2 were each 100 ms	Shallower and longer for Dyslexic's	Shifted to longer durations
	Badcock et al., 2008	Black letters	T1 = white letter	100 ms	No difference between dyslexic and non-dyslexic adults after correcting for baseline sensitivity	No difference between dyslexic and non-dyslexic adults
			T2 = black X			
	Lallier et al., 2010	Black digits	T1 = Red digit (1 or 5)	50 + 66 ms ISI	Lower detection for dyslexics at lag 4. However, no difference between dyslexic and controls. When using technique by Cousineau et al. (2006) Participants had to achieve a performance criteria	No difference between dyslexic and controls.
			T2 = black “0”			
	McLean et al., 2010	1 of 4 arrows	A shape (square, cross, plus, diamond, circle, triangle)	26 + 80 ms ISI	Demonstrated an overall deficit for dyslexic children compared to controls that was not specific to any of the AB parameters	
**Visible persistence**		**Task**	**Details**		**Results**	
	di Lollo et al., 1983	Gap-detection	Line stimulus-gap-line stimulus. Duration of line = 20 ms		Dyslexic readers required longer ISI’s to make accurate judgements to detect the gap between line stimuli	
			Gap ISI = staircase threshold.			
			Participants compared this to a “no-gap” stimulus. The task was to indicate which stimulus contained the gap			
		Pattern integration	Participants were to detect the presence of a missing dot over successively presented dots that form a matrix pattern		No difference between dyslexic and non-dyslexic participants	
	Badcock and Lovegrove, 1981; Slaghuis and Lovegrove, 1985	VP	grating-blank-grating sequence.		Duration of VP was the duration at which the blank field was just visible.	
			Duration of gratings = 300 or 75 ms ISI blank period = staircase threshold.		Dyslexic readers required longer durations to detect the blank period	
			Participants to indicate if they had seen the blank period			
	Slaghuis and Ryan, 1999	Ternus apparent motion	3 squares in a row where the outermost square jumps from the left to the right side. Perception reflects the square jumping (element motion), or all 3 squares moving from left to right (group motion) 40 ms stimulus duration, 10-70 ms ISI. Participants were to indicate whether they saw “group” or “element” movement.		Dyslexic participants were less likely to perceive “group” movement.	
					Suggesting that Dyslexic children demonstrated longer visible persistence	
			120 ms stimulus duration		No differences between groups on the perception of group or element movement	
	Conlon et al., 2004	Temporal counting	Adult dyslexic were required to count the count square targets presented as a RSVP		Dyslexic participants were significantly less accurate in counting rapidly presented stimuli compared to normal adults readers	
	Schulte-Körne et al., 2004	VP	grating-blank-grating sequence.		Duration of VP was the duration at which the blank field was just visible.	
			Duration of gratings = 300 ms		Dyslexic readers were no different from dyslexic readers (indeed normal readers required longer durations to detect the blank period)	
			ISI blank period = staircase threshold.			
			Participants to indicate if they had seen the blank period			
	Jones et al., 2008	Ternus apparent motion	3 squares in a row where the outermost square jumps from the left to the right side. Perception reflects the square jumping (element motion), or all 3 squares moving from left to right (group motion) 40 ms stimulus duration, 10-70 ms ISI. Participants were to indicate whether they saw “group” or “element” movement.		No difference between dyslexic and non-dyslexic children	
**Temporal order judgement**		**Task details**			**Results**	
	Brannan and Williams, 1988	3-letter words, or a symbol (and or #) were presented to the left or right of fixation.			Dyslexic readers required a significantly longer ISI to make accurate judgements regarding which order the stimuli appeared.	
		Stimuli = 900 ms				
		ISI = variable staircase to achieve 75% threshold				
	Hari et al., 2001	Stimuli were presented in the left and right hemifield, participants were to indicate which stimulus appeared first. The ISI between stimuli was varied			Dyslexic participants required longer durations to determine which stimulus appeared first. However, results were asymmetric such that they showed a right visual field (left hemisphere) advantage	
	Jaskowski and Rusiak, 2008	Pairs of rectangles where each were presented above/below or left/right of fixation. Participants had to indicate which rectangle appeared first—the left or the right, the top or the bottom. The ISI was varied between the stimuli presentations.			Dyslexic participants generally required a longer interval to make accurate judgements. However contra to Hari et al, there was no left/right asymmetry	
	Liddle et al., 2009	Stimuli were presented in the left and right hemifield, participants were to indicate which stimulus appeared first. The ISI between stimuli was varied. Participants had to indicate whether the left or right stimuli appeared first. In Exp2 Participants had to indicate the shape of the stimuli that appeared first.			d’ for accuracy showed significantly lower sensitivity for temporal order judgements for dyslexic adults compared to non-dyslexic adults. There was no left/right asymmetry	
**Visual search and change detection^a^**		**Task**	**Details**		**Results**	
	Vidyasagar and Pammer, 1999	Visual Search	Conjunction search using shape and color.		Dyslexic children became progressively less accurate compared to normal reading children, in more cluttered arrays	
	Rutkowski et al., 2003	Change detection	4 letters arranged in a square array around the fixation point. Followed by a blank period (250 ms), followed by another 4-letter stimuli arrangement. The stimuli alternated until a response was made.		Dyslexic children required longer presentation times compared to normal readers to determine whether the two 4-letter target stimuli were the same or different.	
	Jones et al., 2008	Visual search	gratings in a circle around a fixation. 1 target + (2, 4, 8, or 16) distractors. Presentation = 100 ms.		Dyslexic children were less accurate over all set sizes except 2 items	
			Target was an off-vertical grating. Distractors = vertical grating			
	Franceschini et al., 2012	Visual search	Children scan left-to-right across lines of stimuli to circle specific targets.		Poor readers made significantly more errors.	
			Children were young, identified as “at risk” at grade year 1.		Search performance predicted later (1 year) pseudoword reading, text reading and letter naming.	
	de Boer-Schellekens and Vroomen, 2012	Visual search	Distractors = Line segments (24 or 48 items)		Dyslexic readers took significantly longer than normal readers to detect the target, particularly at the larger set size	
			Target = horizontal or vertical line			
			The target and distractors changed color dynamically red through green.			
	Tulloch and Pammer, submitted	Visual Search	Stimuli presented on a computer tablet were “game-like” bugs. Participants had to find the target bug always present on the screen (no memory component)		Search results significantly predicted reading rate for a group of children with a large range of reading ability.	

a Not typically considered a temporal task, but here I am considering the possibility of a static display, where the visual temporal quality occurs because of the “shutter-like” extraction of information at fixations as the eye scans across the page.

Conceptualizing visual temporal processing in this mechanical way also allows us to consider visual search and change detection. Here, like reading, the stimulus is static and the temporal information flow through to the visual system is mediated by fixations, saccades, and eye movements. There is a theoretical argument that links visual search to magnocellular/dorsal processing (Vidyasagar, 1998, 1999; Vidyasagar and Pammer, 1999, 2010), and studies have shown that dyslexic readers are worse than control readers at detecting a target in serial search and change detection (e.g., Vidyasagar and Pammer, 1999; Rutkowski et al., 2003; Jones et al., 2008; de Boer-Schellekens and Vroomen, 2012), and serial search predicts later reading ability in pre-readers (Franceschini et al., 2012).

Thus, many visual temporal coding tasks also draw on quite significant cognitive abilities, such as sequencing, judgment and memory, and although such mechanisms are also required in reading, it makes it difficult to use tasks such as these to explicitly isolate the neural components of visual coding in the temporal sampling process. Studies looking directly at the neural correlates of simple visual synchronisation and entrainment have not been done for normal or dyslexic readers, but would provide a good test of a visual example of the temporal coding hypothesis as has been developed in the auditory domain.

One potential confound for the proposal that visual oscillatory activity may be able to entrain to the temporal sampling rate when reading, is that entrainment or synchronisation is most easily studied in the context of a regular periodicy in the signal stream (refer to Figure 1A). An input signal that does not have a regular periodicy will not generate stable oscillations, because there simply is not a stable oscillatory signal to entrain to (refer to Figure 1D). Reading (like speech perception), is in fact highly variable. Smooth, effortless reading does involve spatial sampling at approximately the theta range, but the crucial word here is “approximately.” Fixations vary considerably: they are longer and more frequent for more difficult words, or unexpected words, regressions are common, and saccade length can also determine the speed at which the text is sampled (refer to Rayner, 1998 for a review), and there are also cognitive confounds such as attention, anticipation, and decision making that could generate their own entrained signals (Thut et al., 2011) which could make it difficult for the system to entrain to the visual input signal. Similarly, in natural language the rhythmicity of the language itself can be quite variable (refer Figure 1E). Despite these reservations, entraining of theta signals to speech *has* been demonstrated (e.g., Luo and Poeppel, 2007), and the theta synchronisation was correlated with speech intelligibility. Furthermore, it is certainly possible that different oscillatory networks may entrain to different frequency components of the speech signal, and poor reading and/or language processing could result from differential impairments in specific frequencies in the overall spectral network, or the interaction of these networks.

If reading *can* be demonstrated to entrain a low-frequency oscillation in the visual domain as a consequence of temporal sampling in the reading process (refer to Figure 1C), then this may have enormous implications for dyslexia. Dyslexic readers' eye movements when reading, are dramatically different from those of normal readers (e.g., refer to Rayner et al., 2001 for a review), with more frequent fixations that are longer and less stable, as well as shorter saccades and more frequent regressions. Moreover it has been suggested that dyslexic readers also have problems in achieving stable binocular control (Stein et al., 2000). Therefore, consistent with Goswami (2011), if normal reading can entrain the visual system, then the highly unstable and variable eye movement behavior in dyslexic readers could very well result in a poor oscillatory coding, because of an inability to generate a systematic, rhythmic saccadic rhythm.

## An argument for a temporal sampling hypothesis in vision in dyslexia

Cortical frequency oscillations have long been considered to underlie visual perception (e.g., refer to Pfurtscheller and Lopes da Silva, 1999; Sewards and Sewards, 1999; Singer, 1999, 2009; Tallon-Baudry and Bertrand, 1999; Engel and Singer, 2001, for reviews). However the question of interest here is the role of cortical frequency oscillations in visual processing in the context of dyslexia. Specifically, do dyslexic readers differ from normal readers in oscillatory phase-locking in visual tasks related to reading. The most direct test of whether dyslexic children demonstrate temporal coding deficits in the visual domain, that are consistent with those found in the auditory domain, is to measure cortical frequency dynamics in dyslexic children for visual stimuli, and/or to evaluate cortical frequency dynamics in dyslexic children in the visuo-spatial areas of the brain. No studies have done this in the context of entrainment to a visual stimulus. Almost all research into cortical frequency dynamics in dyslexic readers, has used auditory stimuli, such as tones (Nagarajan et al., 1999; Ucles et al., 2009; Heim et al., 2011), sounds—such as white noise (Lehongre et al., 2011; Hämäläinen et al., 2012), phonological tasks (Rippon and Brunswick, 2000), or sentences (Han et al., 2012). One EEG study looking at visuo-spatial cuing in dyslexic adults found reduced coherence in the parietal cortex in dyslexic compared to normal readers (Dhar et al., 2010), however the results were only presented in the 8.5 Hz (alpha) range, and it is not known if differential impairments exist in other frequency ranges.

Some studies of linguistic processing in dyslexic readers have used visually presented words, which might allow the extraction of some vision-specific coding. Milne et al. (2003) found an increase in beta power in the posterior brain regions for dyseidetic dyslexic adults, and the reverse pattern, with an increase in beta signals anteriorly for dysphonetic dyslexic adults in a visual lexical decision task. However, only broad-spectrum beta (12–30 Hz) was analyzed, and the lack of spatial specificity inherent in EEG allowed only “posterior” or “anterior” descriptions of the results. Similarly, using EEG and visually presented words, letters or pseudowords, power changes have been demonstrated in theta (Klimesch et al., 2001a,b), as well as alpha and beta oscillations (Klimesch et al., 2001a,b) in dyslexic children. In the first study, differences in theta activity between dyslexics and controls were interpreted to result from difficulties in working memory encoding in dyslexic readers, and potentially more effortful coding for words in occipital sites. In the second study, changes in the patterns of alpha and beta activation were interpreted to reflect differential allocation of sustained attention in dyslexic compared to normal readers. However, in both studies the analysis period consisted of 5 sec after the presentation of a stimulus, making it unlikely that vision-specific coding of the stimuli could be specifically extracted. Other researchers have also presented word, or word-like stimuli visually, but their analyses have been in terms of the linguistic and not the visual coding of the stimuli such that ROI's are language-related areas rather than visual areas (e.g., Spironelli et al., 2008).

To summarize, while a number of studies have specifically looked at oscillatory activity in the auditory cortex of dyslexic readers, none has looked at oscillatory activity to visual temporal coding in the visual cortex of dyslexic readers. This provides two areas to explore: First, in the normal reading population, what is known about cortical oscillatory responses in vision and reading? Second, what is known about cortical responses in some of the visual tasks underlying reading? From these two explorations, a reasonable theoretical framework might be constructed of a role for temporal visual coding deficits in dyslexia.

The visual aspects of reading involve a number of complex and interrelated components, such as object recognition, visual search, extracting information from a cluttered array, guiding eye movements, feature binding, attentional shifting, and preattentive visual coding. Such tasks have been hypothesized to be mediated by the dorsal/magnocellular visual pathway (Vidyasagar, 1998, 1999, 2004), and many of these tasks involve synchronous gamma oscillations (refer to Sauve, 1999; Tallon-Baudry and Bertrand, 1999; Engel and Singer, 2001; Singer, 2009; Tallon-Baudry, 2009; Merker, 2013). Thus it is not at all unreasonable to predict that oscillatory gamma signals are apparent in reading in the way that they are in the perception of speech, and it remains to be seen whether differences in gamma oscillations also characterize dyslexia in the visual domain, like in the auditory domain.

### Temporal sampling and the magnocellular pathway

Given that a deficit in the magnocellular pathway appears to be found in many dyslexic readers, it would seem logical to ascertain whether the magnocellular pathway is selectively responsive to particular cortical frequencies.

Sewards and Sewards (1999) speculated that low frequency oscillations are characteristic of the parvocellular pathway, and high frequency gamma oscillations of the magnocellular pathway. They reached this conclusion from the following evidence: alpha signals are characteristic of stimuli that are static, and where the eyes are also static, whereas gamma *and* alpha oscillations occur when the stimulus itself moves or, with static stimuli when the eyes move. This is consistent with the observation that the magnocellular pathway is responsive to motion. Similarly, cell layers in the LGN that oscillate in the gamma range were found in the layers innervated primarily by retinal magnocellular cells (Livingston, 1996). However, Sewards and Sewards, also point out that Livingstone only measured gamma oscillations, making it difficult to assess the nature of other frequency oscillations in the LGN layers^1^. Sewards and Sewards also suggest that the physiological responses of the magnocells and parvocells are consistent with differential oscillatory activity, in that the fast magno cells are sensitive to temporal frequencies up to 50 Hz, but the slower-transmitting parvo cells have specific modulation frequencies of <10 Hz (Wiesel and Hubel, 1966). More recently, Fründ et al. (2007) demonstrated that low spatial frequency visual stimuli (1 cpd)—which would be transmitted primarily by the magnocellular pathway—generated an evoked gamma signal, whereas high spatial frequency stimuli (10 cpd)—which would be transmitted primarily by the parvocellular pathway—generated an evoked alpha signal.

Pammer et al. (2006) conducted an MEG study in which participants were to perform a lexical decision task that used words presented normally, and words in which the internal letters were shifted up or down relative to each other. The aim was to selectively activate the magno/dorsal pathway in reading, by manipulating the spatial configuration of the internal letters of the words. In this study we demonstrated strong gamma and alpha signals for the “shifted-words” condition, in the posterior parietal cortex—where we would expect magnocellular/dorsal activity. In addition, the time course of these signals was interesting, in that the first signal at approximately 150 ms was a gamma (35–40 Hz) signal, the second, at approximately 200 ms, an alpha signal, and the next signal at about 300 ms, again a gamma signal. It was argued that the initial gamma signal reflected a transient evoked signal—perhaps reflecting initial attentional mechanisms for spatial selection (Moran and Desimone, 1985; Motter, 1993; Vidyasagar, 1998, 1999; Martinez et al., 1999) and that the later gamma signal could be consistent with feature binding (Galambos, 1992; Başar-Eroglu et al., 1996; Pulvermüller et al., 1997; Tallon-Baudry and Bertrand, 1999; Hermann et al., 2004). The alpha signal that occurred between the early and late gamma signals was also associated with activation in a number of different cortical areas, possibly reflecting the recruitment of other cortical areas to support a relatively complex cognitive task.

Although projections of magnocellular and parvocellular neurons through the dorsal and ventral pathways respectively are not completely discrete, making it more difficult to isolate the two pathways, the evidence nevertheless suggests that high frequency neural oscillations could be a mechanism for binding the visual qualities of text, carried by the magnocellular/dorsal pathway. Thus a story is emerging that both high and low frequency signals may be important in visual coding in reading.

### Temporal coding, vision, and reading

**Figure 2:**
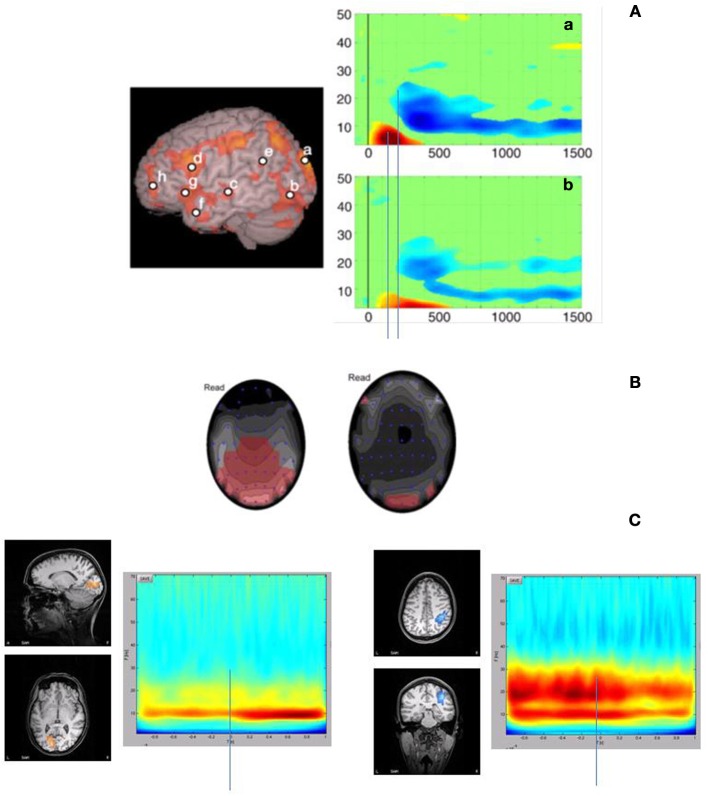
**Some illustrations of the potential relationship between low frequency and high frequency synchronisation in reading. (A)** is adapted from Goto et al. (2011) demonstrating similar ERS and ERD interactions in the theta and gamma ranges in the visual and auditory cortices, refer to text for more detail **(A)** is partially reproduced with permission from Goto et al. (2011, p566, Elsevier). Similarly, Fitzgibbon et al. (2004) also demonstrate increases in gamma power (left) as well as theta (right) **(B)**. **(B)** is partially reproduced with permission from Fitzgibbon et al. (2004, p1806, Elsevier). The findings in **(A)** and **(B)** are consistent with results from our lab in which we demonstrate both gamma and theta/alpha signals to a RSVP reading task **(C)**. In this task, the stimuli were 8–9 word sentences presented RSVP at a rate of 102 ms per word, with a 16 ms ISI. Data was analyzed individually using SAM statistical mapping. SAM is a linear beamforming technique in which the MEG signal is passed through each channel, modified as a weighted linear function of the remaining channels (Vrba, 2002). SAM generates a statistical map by comparing and “active” period with a “control” period. In the current study, the reference control period for all comparisons was 1000 ms prior to the sentence onset. Montages were created for 5–40 Hz frequency bands, and the time × frequency spectrograms were created for two regions of interest that were apparent in all 10 subjects: the right PPc and the visual cortex. In the spectrograms, time includes the 1 sec pre-stimulus interval, and 1 sec sentence duration. Sentence onset is at 0 ms.

Very little research has been done to measure frequency oscillations specifically targeting the visual cortex in reading, and there is a tension between evidence for a theta deficit in dyslexia, and evidence that the magnocellular pathway is associated with gamma oscillations. However a few studies have found vision-specific coding to visually presented words and sentences, in the context of whole-cortex language networks. The results appear to be mixed, but suggest an interesting relationship between gamma and theta oscillations. In one study, Goto et al. (2011) conducted a time × frequency analysis for the reading network. In this study, participants engaged in a silent reading task, and the data was analyzed in five frequency bands (theta, alpha, beta, low gamma, and high gamma), using a 200 ms moving window technique up to 1550 ms post stimulus onset (see also Pammer et al., 2004 for a description of the methodology and an example of the reading network). Figure 2A, is adapted from Goto et al. (2011) and shows two spectrograms of interest. The first (a) is isolated in that part of the visual cortex responsible for the fast coding of the visual components of text. The associated spectrogram suggests that there is an early, transient increase in power (ERS) in the theta range, followed by a sustained decrease in power (ERD) in the beta and low-gamma range. If we map this to a similar spectrogram but in the auditory cortex (in fact the location of “c” in Figure 2 is slightly below the auditory cortex), there is a similar theta ERS approximately 100 ms after the signal in the visual cortex, and then a similar sustained beta/low gamma ERD. It is possible that these signals may be functionally coupled, but this remains to be seen. Moreover, this visual theta signal is also transient, and may reflect quite different functionality to the sustained theta signals described in the auditory temporal coding hypothesis. For example, Bosman et al. (2010) using a visual search and detection task, found that a transient increase in visual theta was locked to stimulus onset when the stimuli was present, but sustained when the target was not present. It was suggested that the sustained response might reflect a sustained search, while the transient response might reflect a stimulus-locked, motor preparation. Thus it will be important to differentiate between transient and sustained signals in terms of functional consequence.

Similarly, we presented participants with nine-word sentences as a rapid serial visual presentation (RSVP) (*Pammer and Holliday, unpublished data*) and found a sustained theta signal. In this study we isolated two visual signals: one in the primary visual cortex, and the other in the posterior parietal cortex (Figure 2C). The former we considered to represent early visual coding, while the latter should involve the dorsal visual pathway and early attentional allocation (e.g., Shomstein and Behrmann, 2006). Like Goto et al., the activation in the primary visual cortex reflects ERS and occurs in the theta/alpha range, start very early, but in this case is sustained. It is likely that this sustained signal reflects the fact that the RSVP were word strings forming sentences, rather than single words as presented in Goto et al. However, whether this signal reflects a “higher-order” cognitive component such as memory, or is a simple visual entrained signal, remains to be seen. With respect to the activity in the posterior parietal cortex, again there was a fast, early theta/alpha signal, but there was also a high frequency gamma signal peaking at approximately 200 ms and then sustained for approximately 500 ms. These results are also consistent with Fitzgibbon et al. (2004), who found both theta and gamma signals in a naturalistic silent reading task. The increase in power relative to a control condition is in red (Figure 2B), and although this was an EEG study and thus lacks some temporal and spatial specificity, the signals are clearly concentrated in the occipital cortex. Other reading studies that have focused on particular frequency bands have also found significant gamma and theta signals (e.g., Bastiaansen et al., 2010) in visual areas in the context of the larger reading network.

In summary, an important and interesting consistency between these studies and the studies in the auditory domain is the presence of oscillatory signals in the theta and gamma range. Thus a coherent framework is beginning to develop that may link poor reading to both theta and gamma synchrony, and thus may start to provide a resolution of the conflict between a temporal coding deficit in dyslexia which is believed to be characterized by deficits in the theta range, and deficits in the magnocellular pathway, where the magnocellular pathway has been implicated in gamma oscillations.

## Physiological mechanisms

The cortical networks involved in reading are highly complex, requiring a sophisticated interplay of temporally and spatially dynamic interactions. Thus, a common mechanism such as temporal sampling for normal reading, and by extension—abnormal reading, could be explained in two possible ways. On the one hand, auditory and visual signals—both necessary for successful reading—may involve unique and discrete phase-locking at the local level, such that deficits within one or both modalities may manifest as a reading difficulty. Another possibility is the importance of long distance cross modal entrainment.

### Cross-modal modulation of oscillatory signals

One way in which cross modal modulation may manifest, is that the gamma and theta signals that are characteristic of language processing in dyslexia and implicated in visual coding in reading, are functionally coupled. One possibility is that the amplitude and/or phase of gamma oscillations are modulated or controlled by the phase of a theta oscillation (Siegel et al., 2012). The implications then are that a theta signal may provide a common regulatory mechanism for higher frequency signals in large-scale cortical networks. This makes intuitive sense in that any large scale neural network such as language perception, memory or reading requires a complex interplay of brain signals from disparate areas in the brain; communicating dynamically by various feed-forward and feedback interactions, with local networks synchronizing at different frequencies both within the network and between individual networks. That a lower-frequency signal may couple to these higher-frequency interactions provides a putative regulatory mechanism that would assist in guiding and adjusting phase and/or amplitude oscillations (e.g., Jensen and Colgin, 2007; Sauseng et al., 2008) between and within local neural networks.

Using direct sub-dural recordings during a number of different perceptual and cognitive tasks, Canolty et al. (2006) demonstrated that the power of very high frequency gamma oscillations (>75 Hz) as modulated by theta signals. Similarly, Doesburg et al. (2012) in an auditory cue task demonstrated significant increases in gamma synchronisation between the auditory and parietal cortex that were modulated by the phase of a concomitant theta signal. However, in both cases, these results could be the result of either within-modality, or within-site coupling. On the other hand, Demiralp et al. (2007) found theta/gamma coupling in a visual perception task, where the amplitude of an occipital gamma synchronisation was associated with a theta occipital as well as a frontal signal, suggesting the existence of more long range theta-to-gamma modulation.

There is also evidence for cross modal oscillatory phase setting such that an auditory signal entrains an oscillatory signal in the visual cortex, and importantly, increases perception for a visual signal (Romei et al., 2012). Although this was only demonstrated for alpha oscillations, it provides a principle to support the possibility that sensory stimuli, for example in the auditory domain, can entrain signals in the visual domain. This is important when considering reading, because the reading process is multi-modal. Behaviorally, reading and word recognition invariably generate a verbal code, and often an internal dialogue, and neuroimaging models of the reading network are consistent in involving visual, auditory and higher order language areas (Pammer et al., 2004).

If theta oscillations assist in coalescing discrete gamma oscillations into large-scale neuronal networks (Siegel et al., 2012), then the abnormal theta oscillations observed in language processing in dyslexia, may have an additional impact in the modulation of gamma synchronisation in the visual domain. Moreover, the abnormal theta oscillations observed may not be functionally important *per se* for the auditory coding of speech, or the visual processing of text, but instead modulate the higher frequency signals required in both the visual and auditory domains. For example, in reading and/or language, a gamma signal may be important for the fast sampling and coding of sensory events at the local level—in the visual domain such sensory coding may be controlled by the magnocellular pathway, while low frequency oscillations like theta may be responsible for more cognitive functions that are ubiquitous across modalities, such as working memory and/or attention, reflecting longer range interactions that mediate cognitive functions.

Lisman and Idiart (1995) were the first to propose that an interaction between low frequency brain waves such as theta oscillations and high frequency gamma oscillations may support short-term or working memory. Sensory—specific information may be coded at high frequencies, in spatially discrete networks (the visual or auditory cortex for example); then bound together in a short-term working memory mechanism coded through low frequency theta waves and via more long range networks, possibly via frontal and/or thalamic networks (refer to Jutras and Buffalo, 2010 for a review). For example, theta-gamma coupling is associated with working memory capacity (Moran et al., 2010), may be particularly important in maintaining temporal rather than spatial relationships (Roberts et al., 2013) and may specifically reflect retention in STM rather than encoding or recognition (Mizuhara and Yamaguchi, 2011).

Another candidate for a top-down theta mechanism, is attention, because both listening to a speech signal and reading require significant attentional input. However the evidence thus far suggests that gamma-theta coupling is specific to working memory, rather than sustained attention (Park et al., 2013). Moreover, attentional modulation of sensory signals appears to be characteristic of gamma and beta signals (refer to Siegel et al., 2012, for a recent review), rather than theta oscillations. Thus, both sensory coding and attentional modulation may be associated with oscillatory activity in the gamma range. It will be important in subsequent research to attempt to isolate attention from sensory coding, before making inferences about functionality. This will be particularly important in attention-intensive tasks like reading.

## Conclusions, questions, hypotheses, and predictions

A substantial amount of research has been devoted to understanding coherent oscillatory activity in dyslexia, but most of this research has focused on the auditory domain. Similar research involving visual processing in dyslexia is virtually non-existent. Thus although I started out with the ambitious aim of describing a visual correlate of the auditory temporal coding hypothesis in dyslexia, this review has in the end attempted to weave together different strands of evidence to support the possibility of a common mechanism for both visual and language deficits in dyslexia. A targeted research program will be necessary to systematically analyse visual coding deficits in dyslexia in the context of temporal coding. Speculations here, suggest a number of hypotheses with clear predictions.

One question reflects the nature of hypothesized visual entrainment to reading. In language, the auditory entrainment occurs via amplitude modulation, but what is the physical visual signal that might entrain a theta rhythm? The first and simplest possibility is that the “shutter-like” quality of the saccade—fixation rhythm projects a rhythmic sequence of patterns at a frequency that will entrain a cortical response. If this was the case, then one should also be able to achieve the same cortical response using a simple RSVP task with patterns, letters or words. However, if cortical entrainment is coupled to the oculomotor behavior of the saccade-fixation rhythm, then maximum entrainment would be dependent on natural, contextual reading. Moreover, if this is the case, then the same signal should be able to be elicited by eye movements that simulate natural reading. Finally, if the cortical signal is in fact dependent on a memory code intrinsic to lexical access and the concatenation of current with previous lexical context when reading, rather than the actual saccade-fixation rhythm, then entrainment should occur in natural reading, but not simulated reading-like eye movements.

The temporal coding hypothesis as applied to auditory processing draws on a large amount of research which has investigated basic auditory coding, and suggests that dyslexic readers are less sensitive to some of the temporal coding aspects of speech, for example amplitude and frequency modulation at low frequencies. A reasonable theoretical framework exists to implicate visual coding in much the same way; the temporal coding of reading mirrors in many ways the temporal coding of speech processing. Therefore, if poor phase locking and entrainment to an external signal forms part of the basis for dyslexia, then we should be able to show responses in the visual domain in dyslexic participants, which mirror those in the auditory domain. For example, if theta synchronisation is pervasive in visual and auditory processing, dyslexic readers should demonstrate reduced sensitivity also in visual tasks that modulate the input frequency in the theta range. For completeness, similar frequency modulations could be done at a range of frequencies in order to explore the possibility of differential sensitivities to different oscillations. Moreover, any sensitivity should also be correlated with reduced auditory sensitivity in the same theta frequency ranges.

Furthermore, exploring a temporal coding framework in other sensory domains from auditory coding, my give us some insight into the discrepancies that exist in the literature, where sensory deficits are not ubiquitous. While discrepant finding are well documented, once again we have little insight into why this might be the case. Thus, as I alluded to above, a temporal coding deficit in dyslexia may in fact be multi-modal, involving auditory, visual, and even motor kinaesthetic (e.g., Thomson and Goswami, 2008) elements. This is consistent with the behavioral literature, and theories suggesting that different manifestations of dyslexia, or different categories of dyslexia may reflect modality-specific differences in sensitivity (e.g., Hogben, 1996; Witton et al., 1998; Stein, 2001; Ramus et al., 2003). Moreover, there is no biological reason why this should not be the case, as multi-modal manifestations of temporal coding are well known, as described above. In the context of dyslexia then, it may be the case that temporal coding deficits may be weighted more heavily toward one or another modality in any individual. Here then, most dyslexic readers may show auditory temporal coding problems of varying degrees, consistent with the relatively stable findings of phonological deficits in dyslexia. However, individuals may also demonstrate visual temporal coding deficits which may manifest as dorsal coding deficits. If this were the case, then we should be able to show that the relative visual, auditory, and even kinaesthetic deficits in temporal coding are directly proportional to the behavioral manifestation of dorsal, auditory, and motor deficits in dyslexia.

Over 20 years of research suggests that there is a deficit in visual coding in dyslexia, and this deficit occurs as a consequence of abnormal visual coding in the magnocellular/dorsal pathway. The evidence reviewed here suggests that the magnocellular visual pathway generates high frequency gamma oscillations. Hence, dyslexic readers may have specific deficits in gamma synchronisation for tasks mediated by the magnocellular pathway, compared to normal readers.

One hypothesis that can be derived from the above discussion is that reading involves binding visual and spatial information in much the same way that occurs for general object recognition. Much of the research on object feature binding implicates gamma frequencies as an important cortical mechanism. Therefore, if dyslexic readers have problems in the binding of visual feature information in a way that is reflected in less stable synchronisation at gamma frequencies, then this should be reflected not only in word recognition, but also in other visual tasks that require feature binding.

The notion that high frequency cortical oscillations are the domain of the magnocellular visual pathway is still relatively speculative. Although good evidence attests to this suggestion, studies are still required to determine the exact spatio-temporal make-up of contrast sensitivity. A logical step here would be a replication of Fründ et al. (2007) but using MEG instead of EEG to allow better spatial mapping of the signals. Like Fründ et al., we would predict sensitivity to stimuli of a low spatial frequency to be associated with signals in the parietal cortex, and brain signals sensitive to higher spatial frequencies to be observed in the inferior temporal areas.

Studies using spatio-temporal mapping such as MEG would allow researchers to attribute oscillatory activity in different areas of the visual cortex to different functional components of the reading network. Such mapping is required at the very least in normal readers, and can then form a framework from which the causes of abnormal reading can be derived.

### Conflict of interest statement

The author declares that the research was conducted in the absence of any commercial or financial relationships that could be construed as a potential conflict of interest.

## Footnotes

^1^It has also recently been demonstrated that low frequency rythms (<10 Hz) are characteristic of cells in the koniocellular layers of the LGN, not the magnocellular or parvocellular layers (Cheong et al., 2011).
